# Governing One Health under climate pressure: policy implementation in Botswana

**DOI:** 10.3389/fpubh.2026.1864265

**Published:** 2026-09-03

**Authors:** Thabo Lucas Seleke

**Affiliations:** Department of Political and Administrative Studies, University of Botswana, Gaborone, Botswana

**Keywords:** Botswana, climate change adaptation, health governance, integrated surveillance, multisectoral coordination, One Health, policy implementation

## Abstract

**Background:**

Climate change is tightening the links between human, animal, and environmental health, especially in settings where systems are already stretched. One Health is now widely recognised in policy circles, but in many cases, it remains more of an intention than something that is consistently practised.

**Methods:**

This paper draws on a qualitative policy analysis of academic literature, national policy documents, and selected comparative examples from Kenya, Bangladesh, and Germany. The aim was not to be exhaustive, but to focus on how implementation unfolds, paying attention to coordination, surveillance, financing, and institutional arrangements.

**Results:**

The main barriers are less related to technical knowledge and more to organisation of systems. Responsibilities are still fragmented, data systems do not easily connect, and financing of joint work is limited. At the same time, there are signs of movement. Efforts around interministerial coordination, integrated surveillance, and regional collaboration suggest that implementation is possible, but uneven and still taking shape.

**Conclusion:**

The findings point to a clear message. Making One Health work in practice depends on how governance is designed and sustained. At its heart, it is about clearer roles, better use of available resources and more consistent ways of working across sectors. In Botswana, where climate and health pressures are coming together, the real challenge is not new frameworks but getting existing commitments to work in practice.

## 1 Introduction

Climate change poses a significant and increasing threat to global health systems, ecosystems, and livelihoods, particularly in low- and middle-income countries (LMICs), where human, animal, and environmental health are closely interconnected (1–3).

Recent evidence shows that climate change is accelerating the emergence and spread of climate-sensitive infectious diseases, antimicrobial resistance, food and water insecurity, and biodiversity loss. These challenges have reinforced the need for integrated governance approaches that cut across traditional sectoral boundaries rather than isolated responses from individual sectors (4–6).

The One Health approach has therefore evolved from a largely conceptual framework into an increasingly important implementation strategy for strengthening surveillance, multisectoral coordination, health-system resilience, and climate adaptation. Although political commitment to One Health has grown considerably in recent years, implementation remains uneven in many LMICs because of fragmented governance arrangements, limited institutional coordination, and inadequate financing for cross-sectoral action (5–8).

This paper examines how integrated health strategies, grounded in the One Health framework, can strengthen responses to climate-related health challenges, with Botswana serving as a policy-relevant case study.

Botswana faces growing exposure to climate-sensitive health threats, including zoonotic disease outbreaks, vector-borne infections, food and water insecurity, and biodiversity loss (9, 10). These risks are expected to intensify as rising temperatures, prolonged droughts, changing rainfall patterns, and ecosystem degradation continue to influence disease transmission, agricultural production, and water security across southern Africa (1, 4, 5).

Drawing on an extensive review of scientific literature, policy documents, and comparative experiences from Kenya, Bangladesh, Germany, and other settings, this paper critically examines the institutional enablers and constraints influencing One Health implementation in Botswana. It identifies key governance challenges, including fragmented institutional mandates, weak data integration systems, limited accountability mechanisms, inadequate financing, and insufficient community engagement.

By situating Botswana within the broader discourse on health governance and climate adaptation, this paper provides practical insights for policymakers, public health practitioners, veterinarians, environmental scientists, and international development partners. More specifically, this paper contributes to health policy and management scholarship by demonstrating that One Health should be understood not only as a technical or interdisciplinary approach, but also as a governance framework for managing climate-related health risks in settings characterised by institutional fragmentation and uneven implementation capacity. This perspective reflects an emerging shift in the One Health literature, where increasing attention is being directed towards governance, institutional coordination, implementation capacity, and policy integration rather than solely disease control or interdisciplinary collaboration (5, 6, 11, 12).

Using Botswana as a policy-relevant case, the analysis demonstrates how legal mandates, intersectoral coordination, financing arrangements, surveillance integration, and community participation shape the extent to which One Health moves from political commitment to routine governance practice (10, 13). In doing so, the paper responds to growing international calls for implementation-oriented One Health research that explains not only why integrated approaches are needed, but also how they can be institutionalised within routine government systems to strengthen climate resilience and health-system performance (4, 7, 8).

From a policy and systems perspective, these challenges are not only epidemiological or environmental but are fundamentally questions of governance. The persistence of climate-sensitive health risks in Botswana reflects the limitations of sectorally organised institutions, where mandates, budgets, surveillance systems, and accountability arrangements remain fragmented across the health, agriculture, environment, and local government sectors. As a result, the effectiveness of climate–health responses depends less on the availability of technical solutions than on the ability of institutions to coordinate, integrate, and sustain cross-sectoral action. Recent implementation research increasingly shows that the success of One Health initiatives depends on institutional capacity, collaborative governance, effective leadership, and mechanisms that enable organisations to work across traditional administrative boundaries. In many LMICs, weak institutional coordination continues to be a greater constraint than technical knowledge or scientific evidence alone (5, 6, 11).

This shifts the analytical focus from isolated interventions to the design and functioning of governance arrangements that enable or constrain integrated health strategies (10, 14). Accordingly, this paper adopts a governance perspective that examines how institutional design, policy coordination, financing arrangements, and accountability mechanisms influence the routine implementation of One Health within climate-sensitive health systems. This implementation-focused perspective reflects an important direction in contemporary One Health scholarship, which increasingly recognises governance as a central determinant of successful policy implementation rather than simply a supporting institutional function (4, 7, 8, 11).

First, the paper discusses the theoretical foundations and evolution of the One Health approach, with particular attention to its growing importance in the context of climate change. Initially developed in response to zoonotic disease outbreaks, One Health has evolved into a comprehensive framework that integrates human, animal, and environmental health across policy, research, and practice.

In the context of climate change, where disease drivers increasingly cross traditional sectoral boundaries, One Health provides a valuable framework for understanding and responding to complex, interconnected health risks. This paper traces this evolution by examining key policy milestones, conceptual developments, and institutional commitments advanced by global organisations, including the Tripartite Guide (13) and the Quadripartite One Health Joint Plan of Action (7). More recent scholarship shows that the One Health agenda has continued to evolve beyond conceptual integration towards practical implementation, institutional governance, climate adaptation, and health-system resilience. Increasing attention is now directed towards translating policy commitments into coordinated action through stronger governance arrangements, integrated surveillance systems, sustainable financing, and multisectoral collaboration (4–6, 11).

Climate change has therefore expanded the scope of One Health beyond the prevention of infectious diseases to include broader challenges such as ecological degradation, antimicrobial resistance, food insecurity, environmental pollution, and the resilience of health systems under increasing climate pressure (7). This shift reflects a growing international consensus that effective One Health implementation requires not only scientific collaboration but also governance systems capable of coordinating institutions, aligning policies, and sustaining cross-sectoral responses over time (6, 8, 11).

Second, the paper shifts attention to Botswana’s climate–health–governance nexus by examining the country’s specific vulnerabilities and institutional arrangements. Botswana is increasingly affected by climate-related health threats, including malaria, Rift Valley fever, heat-related illness, vector-borne diseases, and food- and water-borne infections (9, 10). Recent evidence suggests that climate variability, prolonged droughts, changing rainfall patterns, and ecosystem degradation are increasing the frequency and complexity of these risks, placing additional pressure on already constrained health, veterinary, and environmental systems (1, 4, 5).

Despite growing policy recognition of these challenges, institutional responses remain fragmented. Ministries and agencies continue to operate largely within sector-specific mandates, limiting routine collaboration across health, agriculture, environment, and local government. National policies acknowledge the vulnerability of communities, particularly women, children, and rural populations, to climate-related health threats. However, recent implementation experience indicates that translating these policy commitments into coordinated action has been slow because of fragmented governance arrangements, limited institutional coordination, weak surveillance integration, and resource constraints (5, 6, 11) These implementation challenges illustrate that Botswana’s One Health agenda is no longer constrained primarily by policy recognition. Rather, the central challenge is strengthening the governance arrangements needed to support sustained multisectoral implementation and climate-resilient health systems.

Third, the paper draws on international case studies to generate practical lessons on how One Health strategies have been implemented in response to climate-related health threats across different institutional settings. From Bangladesh’s community-based early warning systems for climate-sensitive diseases, to Kenya’s multisectoral coordination framework for zoonotic disease preparedness, and Germany’s integrated surveillance system for antimicrobial resistance across human, animal, and environmental sectors, the paper presents practical examples of One Health implementation in action (15, 16). Recent implementation experience demonstrates that there is no single model for operationalising One Health. Rather, countries have adopted different governance arrangements depending on their institutional capacity, health priorities, financing mechanisms, and policy contexts (5, 6, 8, 11).

These case studies provide practical lessons on institutional reform, multisectoral governance, community participation, digital surveillance, and sustainable implementation. They also illustrate how legal mandates, political commitment, leadership, and accountability influence the institutionalisation of One Health beyond pilot projects and short-term initiatives (5, 8, 11). This implementation perspective is particularly relevant for Botswana, where strengthening governance arrangements remains central to achieving routine cross-sectoral collaboration (6, 10).

The comparative analysis therefore positions Botswana within a broader global learning environment and highlights the value of policy learning across both high-income and low- and middle-income countries. Rather than promoting direct policy transfer, the paper uses these international experiences to identify governance principles that can be adapted to Botswana’s institutional context and climate-health priorities (11, 14, 15). Ultimately, the paper aims to provide evidence-based policy and implementation recommendations for strengthening One Health integration in Botswana.

These recommendations target multiple levels of governance, ranging from national institutions and local authorities to regional organisations and international development partners. They emphasise the importance of developing integrated surveillance systems that connect human, animal, and environmental health; strengthening institutional coordination across ministries; embedding One Health within national health, climate, and development policies; and actively engaging communities in the design, implementation, and monitoring of local initiatives (5–8).

The paper also highlights the importance of strengthening governance arrangements that support routine multisectoral collaboration, sustainable financing, interoperable data systems, workforce capacity, and institutional accountability. These elements are increasingly recognised as essential for translating One Health policy commitments into effective implementation (5–8, 11). Special emphasis is placed on financing mechanisms, cross-sectoral data sharing, professional capacity development, and public awareness, all of which contribute to a supportive environment for integrated One Health implementation (6, 8).

Rather than proposing new institutional structures alone, the paper argues that strengthening existing governance systems, improving policy coordination, and embedding One Health within routine government processes are more likely to produce sustainable and climate-resilient health systems. This governance-centred approach is consistent with emerging international evidence showing that long-term institutionalisation depends less on creating new organisations than on strengthening coordination, accountability, financing, and implementation capacity within existing systems (5, 11). In doing so, the paper moves beyond rhetorical commitment and offers practical strategies for institutionalising One Health within Botswana’s broader climate resilience and health-system strengthening agenda.

Together, these objectives provide the foundation for a comprehensive examination of how Botswana and other climate-vulnerable countries can strengthen One Health implementation through integrated governance, multisectoral collaboration, and resilient health systems. More broadly, the paper examines how governance, institutional coordination, and implementation capacity shape the ability of health systems to respond to the interconnected challenges of climate change, health insecurity, and environmental degradation. In doing so, it contributes to the growing body of implementation-oriented One Health scholarship that seeks practical pathways for translating policy commitments into routine governance and sustainable action (4, 8, 11).

## 2 Methods

This paper uses a qualitative policy analysis based on a targeted review of academic and grey literature, supported by comparative case material. The analysis was designed to examine how the One Health approach is translated from broad policy commitment into practical governance arrangements, using Botswana as the primary case study. Rather than generating primary data, the paper draws on existing evidence to examine the institutional, policy, and implementation conditions that shape One Health practice in climate-sensitive settings. The review focused primarily on literature published between 2010 and March 2026, while retaining selected seminal publications published before 2010 where these remained foundational to the development of One Health theory, governance, and health systems research. Particular attention was given to peer-reviewed studies and policy documents published from 2024 onwards to capture recent developments in One Health implementation, climate-health governance, multisectoral collaboration, and health-system resilience.

The review combined three categories of material. First, it examined peer-reviewed literature on One Health, climate change, health systems, multisectoral governance, integrated surveillance, climate adaptation, antimicrobial resistance, and implementation research. Second, it reviewed policy and institutional documents relevant to Botswana, including national health, climate, and development frameworks, together with selected publications from international organisations such as WHO, FAO, WOAH, UNEP, the World Bank, the African Union, and Africa CDC. Third, the analysis drew on comparative experiences from Kenya, Bangladesh, Germany, and regional African initiatives to identify practical lessons on governance, surveillance, financing, and institutional design.

The selection of documents was purposive rather than exhaustive. Sources were included if they addressed one or more of the following themes: One Health governance, climate-related health risks, intersectoral coordination, surveillance integration, financing arrangements, implementation barriers, health-system resilience, or policy learning relevant to Botswana. The comparative cases were selected because they illustrate different approaches to operationalising One Health in practice. Kenya provides lessons on multisectoral coordination for zoonotic disease preparedness in an African context. Bangladesh demonstrates the value of climate-linked and community-based surveillance. Germany illustrates a more institutionalised model of multisectoral governance, particularly in antimicrobial resistance. These cases were not treated as directly transferable models but as practical reference points for identifying governance lessons relevant to Botswana.

The material was analysed thematically. The analysis was guided by recurring themes identified across the literature and policy documents, including institutional fragmentation, legal mandates, coordination mechanisms, surveillance integration, data sharing, financing, workforce capacity, accountability, and community participation. Particular attention was given to identifying governance arrangements that either enabled or constrained routine implementation of One Health across different institutional contexts. The analysis was further informed by a health policy and systems perspective, examining how responsibilities are distributed across sectors, how implementation is facilitated or constrained, and how policy commitments are translated into routine practice. This approach enabled the analysis to move beyond descriptive synthesis and focus on the institutional conditions that support or hinder effective One Health implementation (17, 18).

This methodological approach is appropriate because the purpose of the paper is not to measure programme outcomes statistically but to examine policy instruments, institutional arrangements, and implementation experiences in settings where One Health remains unevenly institutionalised. The study nevertheless has limitations. Because it relies exclusively on secondary sources, it cannot capture the day-to-day experiences of policymakers, practitioners, or frontline implementers. The paper therefore presents an analytically informed policy interpretation rather than an empirical evaluation based on primary data. Even so, the combination of peer-reviewed literature, policy documents, and comparative case studies provides a robust foundation for examining how One Health can be more effectively institutionalised in Botswana under conditions of climate change and increasing health-system vulnerability.

## 3 One Health: conceptual foundations and contemporary relevance

The One Health framework represents a fundamental shift in how health systems are understood. Rather than viewing human, animal, and environmental health as separate domains, it recognises them as interconnected components of a single system that requires coordinated governance and action. At its core, One Health acknowledges the close interdependence between human, animal, and environmental health, an insight that has become increasingly important in the Anthropocene, where ecological disruption, climate change, global mobility, and emerging zoonotic threats are reshaping health risks (12, 13). Recent scholarship has further expanded this perspective by emphasising that One Health is not only a scientific or interdisciplinary concept but also an implementation framework that supports integrated governance, climate adaptation, and resilient health systems. Increasing attention is therefore being directed towards the institutional arrangements needed to translate One Health from policy commitment into routine practice (4–6, 11). Practically, One Health challenges fragmented planning and promotes systems thinking, transdisciplinary collaboration, and coordinated responses to interconnected risks ranging from zoonotic spillovers to climate-related health emergencies.

### 3.1 Conceptual origins and evolution

Although often described as a contemporary approach, the One Health paradigm is rooted in a long tradition of interdisciplinary thinking. During the nineteenth century, Rudolf Virchow, who coined the term *zoonosis*, argued that there should be no distinction between human and animal medicine. Around the same period, William Osler promoted comparative approaches that linked pathology across species (19). These early ideas influenced later developments in veterinary medicine, food safety, and rabies control. However, collaboration remained largely informal because institutional mandates, governance structures, and professional systems continued to operate independently.

Momentum shifted around the turn of the twenty-first century as successive disease outbreaks exposed the limitations of sector-specific responses. Outbreaks of H5N1 (1997), SARS-CoV-1 (2002–2003), and H1N1 (2009) demonstrated that emerging infectious diseases frequently arise at the human–animal–environment interface and require coordinated surveillance and preparedness across sectors (13, 20). A major milestone was the adoption of the Manhattan Principles in 2004, which called for ecosystem-based approaches to disease prevention and recognised One Health as both a scientific framework and a governance approach that promotes collaboration across human, animal, and environmental health (21).

From this point, institutionalisation accelerated. The FAO, OIE, and WHO Tripartite collaboration produced joint guidance, supported national One Health strategies, and developed coordinated approaches for zoonotic diseases, rabies, avian influenza, and antimicrobial resistance (13). The inclusion of UNEP in 2022 expanded this partnership into the Quadripartite and resulted in the One Health Joint Plan of Action (2022–2026). The Plan broadened the scope of One Health beyond zoonotic diseases to include climate change, biodiversity loss, food safety, ecosystem degradation, and sustainable development. It also emphasised systems thinking, community participation, integrated surveillance, workforce development, and sustainable financing (7).

Since the adoption of the Joint Plan of Action, attention has increasingly shifted from developing One Health policies towards strengthening their implementation. Recent international guidance highlights the importance of institutional leadership, multisectoral governance, interoperable surveillance systems, workforce capacity, and sustainable financing as essential requirements for translating policy commitments into routine practice (5, 6, 8, 11). This shift reflects a broader recognition that effective One Health implementation depends as much on governance and institutional capacity as on scientific and technical expertise (11, 12).

One Health has also become increasingly embedded within global health security and sustainable development agendas. The International Health Regulations (2005), the Global Health Security Agenda, and the Sustainable Development Goals collectively recognise the importance of multisectoral collaboration in preventing and responding to health threats (20, 22). Regional interpretations nevertheless differ. High-income countries have often approached One Health through the lens of biosecurity and pandemic preparedness. In contrast, many low- and middle-income countries, particularly in sub-Saharan Africa, increasingly view One Health as a framework for strengthening health systems, promoting climate resilience, and supporting integrated development where environmental degradation, poverty, and health risks converge (5, 7, 19).

More recent African experience further illustrates this transition. Countries are increasingly embedding One Health within national governance structures, recognising that effective implementation requires sustained political commitment, institutional coordination, and long-term investment rather than short-term project-based interventions (5, 8, 11).

One Health has therefore evolved from a collection of interdisciplinary ideas into an internationally recognised governance framework supported by multilateral institutions and increasingly reflected in national and regional policies. The next challenge is not conceptual development but implementation. For countries such as Botswana, this means translating One Health principles into governance arrangements that strengthen institutional coordination, climate resilience, and health-system performance within routine government practice.

### 3.2 Key principles and strategic pillars

The One Health approach is grounded in a set of interconnected principles that reflect both its philosophical foundations and practical objectives. These guiding values, including systems thinking, transdisciplinary collaboration, equity, inclusivity, evidence-based practice, and sustainability, serve as the normative and operational basis for designing and implementing One Health strategies. Each principle helps shape how institutions, communities, and professionals collaborate to address the complex interconnections between human, animal, and environmental health, particularly in an era of climate change and ecological vulnerability.

Recent international guidance has reinforced these principles by placing greater emphasis on implementation, institutional coordination, climate resilience, and health-system strengthening as essential components of effective One Health governance (5, 6, 8). At its core, One Health is based on systems thinking, which suggests that health outcomes do not result from isolated factors but from complex interactions across different sectors and levels of governance. This systems approach encourages policymakers and practitioners to examine how land use, livestock practices, urbanisation, water systems, and biodiversity loss are interconnected with disease outbreaks, food security, and overall population health. As a result, systems thinking challenges simplified models of disease control and supports comprehensive, multi-faceted solutions that target root causes rather than symptoms. Increasingly, systems thinking is also being applied to climate-health planning because it enables policymakers to understand how environmental change, institutional arrangements, and health outcomes interact across sectors rather than in isolation (4, 12).

Complementing systems thinking is the principle of transdisciplinarity, which extends beyond interdisciplinary collaboration to encompass diverse stakeholders, including scientists, policymakers, local communities, traditional leaders, and civil society actors, in the co-creation of knowledge and solutions. This approach recognises that complex health challenges, such as zoonotic spillovers or antimicrobial resistance, cannot be solved by any single discipline or sector alone. Recent implementation research further shows that transdisciplinary governance strengthens institutional coordination, improves policy coherence, and facilitates more sustainable implementation by integrating scientific, community, and policy perspectives into routine decision-making (8, 11). Transdisciplinarity promotes mutual learning and trust, helping stakeholders align priorities and share resources across institutions.

Equity and inclusivity are equally fundamental to One Health. These principles emphasise the importance of ensuring that One Health strategies are designed and implemented in ways that address social and economic inequalities, particularly among vulnerable populations. In many LMICs, marginalised groups such as rural pastoralists, women in subsistence farming communities, and Indigenous peoples bear a disproportionate burden of climate-related health risks. An equitable One Health approach ensures that these communities are not passive recipients of top-down interventions but active participants in designing, implementing, and monitoring programmes that affect their lives. Inclusivity also involves recognising and valuing Indigenous knowledge systems, which often provide detailed insights into ecological cycles, animal behaviour, and environmental change that support early warning and locally appropriate responses. This emphasis also aligns with growing international recognition that climate adaptation and One Health implementation should be guided by principles of equity and climate justice, ensuring that vulnerable communities benefit from integrated policies and investments (5, 8, 12).

Another defining attribute of One Health is its commitment to evidence-based practice. This principle emphasises the importance of generating rigorous, timely, and contextually relevant evidence to inform decision-making and monitor progress. Within the One Health framework, evidence should be drawn from multiple disciplines, including epidemiology, ecology, sociology, and veterinary science, and shared transparently across institutions. Effective decision-making therefore depends on integrating information across human, animal, and environmental health systems. Recent implementation guidance further highlights the importance of interoperable digital information systems, integrated surveillance platforms, and timely cross-sector data exchange for supporting evidence-informed decision-making and coordinated responses (5, 6, 8).

These guiding principles are operationalised through a set of strategic pillars that translate normative goals into practical action. One of the most important pillars is the development of integrated surveillance and early warning systems capable of detecting health threats across sectors. In the context of climate change, where disease vectors are changing geographically and seasonally, surveillance systems that integrate veterinary information, human health records, and environmental indicators are increasingly important. Monitoring mosquito breeding habitats, livestock mortality, and patterns of human febrile illness together can provide early warning of outbreaks such as Rift Valley fever and malaria (4, 23). Advances in digital surveillance, geographic information systems, and predictive analytics are further strengthening countries’ capacity to anticipate climate-sensitive health threats before they develop into major public health emergencies (5, 6).

Equally important is the establishment of integrated governance structures that facilitate coordination among ministries responsible for health, agriculture, environment, and local government, together with academic institutions, civil society organisations, and international partners. Without effective coordination, interventions may be duplicated, resources fragmented, and important risks overlooked. Integrated governance promotes shared accountability, clarifies institutional responsibilities, and supports more efficient use of limited resources. Recent implementation experience demonstrates that successful One Health governance depends not only on coordination mechanisms but also on clear institutional mandates, sustained political commitment, predictable financing, and joint accountability across sectors (5, 6, 8, 11).

A third strategic pillar is cross-sectoral data sharing and analysis, which supports the integration of information from multiple sectors into actionable intelligence. Effective One Health implementation requires breaking down the silos separating disease surveillance databases, agricultural information systems, land-use monitoring, and climate datasets. Establishing interoperable platforms, standardised indicators, and joint analytical processes enables a more comprehensive understanding of emerging risks and supports evidence-informed decisions on outbreak response, climate adaptation, and resource allocation (5, 6, 8).

In addition to infrastructure and information systems, the One Health approach requires sustained workforce development across human, animal, and environmental health professions. This includes training veterinarians, public health professionals, environmental scientists, and frontline workers in integrated surveillance, joint investigations, and interprofessional collaboration. University curricula and professional development programmes should also promote systems thinking and practical field experience in implementing One Health (5, 8).

Countries that invest in a multidisciplinary One Health workforce are better positioned to identify emerging threats, coordinate responses, and strengthen long-term resilience (5, 8).

Finally, the success of any One Health initiative depends on meaningful community participation and local ownership. Without community engagement, even technically sound interventions may fail to gain legitimacy or be sustained over time. Participatory approaches such as community scorecards, citizen science initiatives, and inclusive policy dialogues help ensure that interventions reflect local priorities and realities. In many rural communities in Botswana, traditional livestock management practices, Indigenous ecological knowledge, and local social institutions play an important role in shaping how health risks are understood and managed. Integrating this knowledge with scientific evidence improves the cultural acceptability, operational feasibility, and long-term sustainability of One Health interventions. Recent implementation research increasingly recognises communities as partners in governance rather than simply beneficiaries of programmes. Building trust, strengthening local ownership, and promoting co-production of solutions have become central elements of sustainable One Health implementation (One Health High-Level Expert (5, 8, 11, 12)).

In conclusion, the strategic pillars of the One Health framework should not be viewed as isolated mechanisms but as mutually reinforcing components of an integrated governance architecture. When implemented together and guided by systems thinking, transdisciplinary collaboration, equity, and evidence-informed decision-making, they can transform fragmented sectoral responses into coordinated strategies that are more resilient to climate change and other complex health threats. For countries such as Botswana, strengthening these strategic pillars offers an opportunity not only to improve preparedness for emerging health threats but also to institutionalise multisectoral governance, strengthen climate resilience, and build more adaptive, people-centred health systems capable of responding to increasingly interconnected environmental and public health challenges.

### 3.3 One health and climate change: the converging crisis

Climate change and health are now frequently discussed together in global policy debates. Major assessments indicate that human-driven environmental change poses a significant threat to the planet and is also becoming a public health emergency (17, 24, 25). This overlap highlights the limitations of narrow, sector-only responses and strengthens the argument for working through One Health. As climate hazards intensify, they exacerbate the very risks that One Health is designed to manage, making its use both urgent and essential (13).

Increasingly, climate change is also understood as a governance challenge that exposes weaknesses in institutional coordination, policy coherence, and public sector preparedness rather than solely an environmental or health problem (4, 5). Building climate-resilient health systems therefore requires integrated governance arrangements capable of coordinating actions across sectors, levels of government, and disciplinary boundaries. This perspective aligns closely with the One Health approach, which promotes collective action in managing complex risks that no single institution can effectively address.

Warming, erratic rain, spreading deserts, and biodiversity loss are no longer distant issues; they are daily challenges facing people, animals, and ecosystems (1, 3). The Lancet Countdown reports an increase in heat-related illnesses, climate-driven shifts in the ranges of mosquitoes and ticks, and repeated disruptions to food and water systems (24). Diseases once controlled by altitude or latitude, such as malaria, now appear in areas that were not previously endemic; similar patterns are observed with dengue, chikungunya, and Zika (1, 26, 27).

These changes mainly affect regions with fragile institutions (28). In East Africa, prolonged droughts push pastoralists into new areas, increasing interactions among people, livestock, and wildlife, which raises the risk of Rift Valley fever, brucellosis, and anthrax (29). In West and Central Africa, deforestation and habitat loss change human-wildlife contact and have been linked to Ebola outbreaks (1, 3). Floods and storms also help spread water- and soil-borne diseases. In South Asia, floods often cause outbreaks of leptospirosis, cholera, and other waterborne diseases. In Bangladesh, heavy monsoons can increase rodent populations and raise the risk of leptospirosis for both humans and animals (1, 3). When sanitation systems fail, these risks grow even more. These patterns emphasise the importance of integrated surveillance and coordinated public health actions at the human–animal–environment interface (13). In flood- and vector-prone areas, combining environmental monitoring with human–animal sentinel systems improves response speed and accuracy (23).

Climate change may also contribute indirectly to the emergence and spread of antimicrobial resistance (AMR), an issue that has become an important component of the expanded One Health agenda. Rising temperatures, extreme weather events, disrupted sanitation systems, and changes in antimicrobial use in both human and animal populations can facilitate the environmental transmission of resistant organisms. Recognising these interconnected risks, the Quadripartite has increasingly positioned AMR alongside zoonotic diseases, food safety, and environmental health within a unified One Health framework (7).

Climate change also raises indirect risks. Food insecurity, displacement, overcrowding, and resource conflicts, often caused or exacerbated by climate shocks, alter exposure pathways and increase transmission, resulting in reduced harvests, weakened nutrition, forced migration into informal settlements, and conditions conducive to cholera, typhoid, and respiratory illnesses (30). Repeated shocks also stretch health systems and slow detection and response (24). On the governance side, climate hazards transcend boundaries and undermine mandates. Many frameworks remain reactive and sector-specific, built for one-off events rather than linked risks. The result is fragmentation, duplication, and slow coordination. Priorities do not align; data systems do not speak to each other; and joint planning is weak (4, 6, 31).

Recent global discussions increasingly emphasise that climate resilience depends not only on technical capacity but also on institutional arrangements that enable joint planning, interoperable surveillance systems, sustainable financing, and shared accountability across sectors (5, 32). Strengthening these governance mechanisms improves preparedness, supports more efficient resource allocation, and enables coordinated responses before localised threats escalate into broader public health emergencies.

In Botswana, veterinary surveillance remains only partially connected to human public health reporting, which delays the detection and control of zoonotic spillovers (9, 10). Although Botswana has strengthened multisectoral collaboration in recent years, routine integration of surveillance information across the human, animal, and environmental health sectors remains an important implementation priority. One Health provides a practical solution. It unites sectors for shared risk assessment, coordinated plans, and joint actions, enabling authorities to anticipate climate-related threats rather than just respond to them (13). It also offers a clear pathway to integrate climate–health priorities into broader development, disaster risk reduction, and public finance efforts, now reflected in the expanded Quadripartite work (7).

Botswana’s increasing exposure to prolonged droughts, pressure on scarce water resources, and changing interactions between wildlife, livestock, and human settlements further reinforces the need for integrated climate-sensitive surveillance systems. Rather than focusing predominantly on veterinary reporting, future surveillance systems should incorporate environmental, meteorological, wildlife, and human health information to provide earlier warning of emerging climate-related health threats (5, 10).

To make this real, countries need One Health integrated into National Adaptation Plans (NAPs) and Nationally Determined Contributions (NDCs). Many of these documents mention health but fall short of implementing firm, cross-sector actions (1, 3). Omitting One Health weakens resilience. This requires the development of surveillance and early warning systems that detect and respond to health risks across sectors (13). COVID-19 demonstrated how land-use change and global mobility can transform a local spillover into a worldwide crisis (3). As climate change is expected to increase the frequency and severity of such events, One Health expands its focus beyond health and agriculture to include climate finance, development planning, and disaster preparedness (7).

Climate change tests and confirms the importance of One Health. It increases interdependence among humans, animals, and ecosystems; highlights gaps in governance; and requires integrated responses (13). For Botswana, where climate stress overlaps with fragile ecosystems and an evolving public health system, the concept of One Health is vital (10). Implementation will depend on political will, technical capacity, and a mindset that considers health security and environmental stewardship as a unified goal.

Looking ahead, embedding One Health within national climate adaptation strategies, disaster risk reduction frameworks, and sustainable development planning offers an opportunity to build more resilient institutions capable of responding to increasingly complex environmental and public health challenges. As international policy continues to converge around integrated approaches to climate resilience, countries that strengthen multisectoral governance and coordinated implementation will be better positioned to protect both population health and ecosystem sustainability (4, 32).

### 3.4 Institutionalising one health: from concept to routine governance, with lessons for Botswana

The evolution of the One Health paradigm from a conceptual lens to an operational strategy has proceeded through its gradual institutionalisation within national and regional governance systems. Institutionalisation extends beyond rhetorical endorsement to include formal recognition in policies and plans, the creation of standing coordination entities, the establishment of legal mandates for inter-agency action, and the allocation of stable budgets linked to measurable performance. Although levels of maturity vary, experiences from East Africa, Europe, and South Asia demonstrate that translating One Health into practice is feasible where political commitment, workable financing arrangements, and local institutional capacity align (11, 33).

Recent guidance from the Quadripartite and the World Health Organization further emphasises that institutionalisation should be understood as a continuous governance process rather than a one-time policy reform. Sustaining One Health requires mechanisms that survive political transitions, integrate routine planning and budgeting, and embed collaborative practices within the day-to-day operations of government institutions (6–8).

The East African Community (EAC) serves as a key regional example. The EAC Regional One Health Strategy 2022–2027 provides a framework for coordinating surveillance, risk assessment, and outbreak response among member states (34). The plan implemented One Health through National One Health Platforms, which bring together health, agriculture/livestock, environment, and wildlife authorities under standard terms of reference (34). It was supported by regional investments through the World Bank’s REDISSE Programme (35). These platforms proved especially useful during cross-border events such as Ebola alerts and Rift Valley fever by minimising duplication, clarifying leadership roles, and speeding up joint decision-making (8, 34). Kenya’s Zoonotic Disease Unit highlights how regional guidance can be effectively adopted at the national level; its standard operating procedures combine veterinary and public health functions for preparedness, investigation, and post-incident learning (Kenya Zoonotic Disease (36)). Importantly, capacity development was regarded as a core function rather than an extra, with frontline training, reference laboratories, and joint simulation exercises used to foster a collaborative culture (34).

The East African experience also illustrates the value of regional institutions in promoting policy learning, harmonising technical standards, and facilitating mutual accountability among member states. Such arrangements reduce institutional fragmentation by providing common operating procedures while allowing countries sufficient flexibility to adapt implementation to their national contexts (8, 11).

Germany’s experience shows how One Health can be embedded through a specific issue—antimicrobial resistance (AMR). Under the national AMR strategy (DART 2030), institutions like the Robert Koch Institute and the Federal Institute for Risk Assessment coordinate surveillance and risk management across human, animal, and environmental sectors (15, 16). The collaboration is not optional: data sharing, joint working groups, and measurable actions are required by the strategy and backed by steady public funding (16). By explicitly including environmental sectors such as wastewater management, agriculture, and food safety, Germany expanded the One Health approach beyond zoonoses to include broader ecological determinants of health (15, 16). The key lesson is that inter-sectoral work only becomes routine when it has legal backing, predictable budgets, and clear lines of accountability across federal, regional, and local levels (16).

Germany further demonstrates that institutional maturity depends not only on technical expertise but also on governance arrangements that support transparency, routine performance monitoring, and continuous organisational learning. These features strengthen resilience by allowing institutions to adapt to emerging threats without requiring major structural reforms each time new risks arise (11, 15).

Bangladesh presents a different model shaped by climate and infectious-disease risks. Its One Health Strategic Framework established an interministerial governance structure linking human health, livestock, environment, agriculture, and other relevant agencies. The framework prioritises coordinated surveillance, joint outbreak investigation, data sharing, district-level coordination, strategic communication, and workforce development (37). A distinctive feature is its emphasis on extending coordination below the national level and strengthening event-based reporting across human, animal, and environmental health systems. This approach demonstrates how One Health can be adapted to a densely populated and climate-vulnerable setting through formal governance arrangements, coordinated surveillance, and locally grounded implementation (8, 37).

The Bangladesh experience also highlights the importance of community participation as a governance resource rather than simply a service delivery mechanism. Incorporating local knowledge and district-level reporting into surveillance systems can improve early warning, institutional legitimacy, and public trust during health emergencies (8, 37).

Taken together, these cases suggest that effective institutionalisation is less a technical exercise and more an organisational and political redesign. It requires mandates that compel collaboration, budget lines that finance joint work rather than short-term projects, interoperable data systems, and frontline capabilities to detect and act on early signals (8, 11).

For Botswana, these international experiences suggest that future progress will depend less on creating entirely new institutions than on strengthening coordination among existing ministries responsible for health, animal health, wildlife, agriculture, environment, disaster management, and local government. Institutionalising regular joint planning, integrated surveillance, shared financing arrangements, and common performance indicators would move One Health from project-based collaboration towards routine public sector governance (6, 8). Such reforms would also position Botswana to respond more effectively to emerging climate-related health threats while supporting broader national goals of health security, environmental sustainability, and resilient development (5, 10).

#### 3.4.1 Adapting lessons for Botswana

Botswana starts with a solid foundation. It has effective public health and veterinary services, a governance system driven by policies, and increasing abilities in managing environmental and climate risks (10). Transitioning from ideas to everyday practice would benefit from three interconnected steps.

Importantly, institutionalising One Health should build upon existing governance structures rather than creating parallel systems that duplicate responsibilities. International experience suggests that reforms are more sustainable when they strengthen coordination among established institutions while preserving clear lines of accountability and sectoral expertise (7, 8, 11).

First, the country should establish a legally based national platform for One Health coordination. A ministerial directive or legal notice under the Public Health Act could create a National One Health Platform that brings together the Ministry of Health, Veterinary Services within the Ministry of Agriculture, the environmental authority, and disaster management. Placing the secretariat within the Department of Public Health would align One Health with existing surveillance and emergency functions. The founding document should specify mandatory data sharing, joint investigation procedures, and escalation rules. Borrowing from the EAC template, standardised terms of reference would minimise ambiguity, and linking the platform to SADC surveillance mechanisms would ensure regional coherence during transboundary events (8, 34).

The platform should also establish regular joint planning cycles, simulation exercises, and after-action reviews to ensure that collaboration becomes routine rather than being activated only during emergencies. These governance mechanisms promote continuous institutional learning and strengthen preparedness for future climate-related and zoonotic health threats (8, 11).

Second, Botswana should incorporate One Health into its budget and performance systems rather than relying only on project funding. A modest but dedicated budget line within the Ministry of Health could support joint simulations, integrated training, and environmental health monitoring with clear deliverables. Metrics should be included in the national Health Sector Performance Matrix and reported using a traffic-light (green, amber, red) scheme to indicate what is on track, what needs corrective action, and what requires escalation. The logic of Germany’s DART 2030 framework is instructive: collaboration becomes reliable when connected to statutory reporting, shared indicators, and predictable funding (16).

Embedding One Health indicators within existing government planning and budgeting systems would also improve accountability by allowing progress to be monitored through routine national performance reviews rather than stand-alone project reports. This would help ensure that One Health remains a sustained government priority beyond changes in donor funding or political leadership (6, 8).

Third, institutionalisation will rely on a comprehensive local detection network. Botswana can strengthen coordination among its District Health Management Teams, Livestock Advisory Centres, and Village Development Committees to support a coordinated surveillance system that collects community-level signals and feeds them into national decision-making systems. Training community animal health workers and environmental officers to recognise and report “unusual events,” along with simple digital reporting tools and district validation, would shorten the time between signal detection and response (6, 9). As Botswana’s National Public Health Institute becomes operational, it can serve as the national hub for integrating multisectoral data, creating risk maps, and coordinating early warning and response (10).

Advances in digital health infrastructure, geographic information systems, and interoperable surveillance platforms also create opportunities to integrate human, animal, and environmental information into a common decision-support system. Such integration would strengthen early warning capacity while improving the efficiency of national preparedness and response mechanisms (6, 8).

Botswana’s path forward is clear: establish a legally sound platform, fund and oversee joint efforts within the main budget, and integrate community insights into national systems. These reforms would turn One Health from an aspiration into tangible governance improvements that enhance anticipation, coordination, and accountability as climate pressures and shifting disease patterns challenge the health system’s resilience (5, 10).

More broadly, successful implementation will depend on sustained political commitment, adequate domestic investment, and a culture of collaboration across sectors. Institutionalising One Health should therefore be viewed as a long-term governance reform that strengthens health system resilience while contributing to national climate adaptation, disaster preparedness, and sustainable development objectives (5, 8, 11).

To illustrate these lessons, Table 1 presents a comparative overview of One Health implementation in selected countries.

**Table 1:** Comparative overview of One Health institutionalisation and transferable governance lessons for Botswana.

Jurisdiction	Institutional vehicle	Legal/mandate & financing	Stand-out practices	Transferable lesson for Botswana
East African Community (regional)	Regional One Health Strategic Plan; National One Health Platforms	Regional framework; donor-assisted start-up; growing national ownership	Cross-border simulations; joint risk assessment; reference labs; frontline training	Constitute a legally anchored national platform with standard terms of reference and escalation rules; align with SADC surveillance
Kenya	Zoonotic Disease Unit linking MoH and Livestock	Formal unit with SOPs and data-sharing protocols	Integrated preparedness and response; after-action reviews	House coordination in a standing unit with binding SOPs and shared datasets
Germany	RKI–BfR coordination under DART (AMR)	Strategy with legal force; stable public budgets; KPIs and statutory reporting	Human–animal–environment AMR surveillance; wastewater integration; multi-level governance	Make collaboration mandatory and funded; extend beyond zoonoses to environmental determinants
Bangladesh	One Health Secretariat and interministerial framework	Government-led; embedded in disaster/climate operations; mixed financing	Risk-based surveillance; community event-based reporting; district-level coordination	Build bottom-up detection via DHMTs and community reporters; integrate local ecological knowledge

RKI, Robert Koch Institute; BfR, Federal Institute for Risk Assessment; DART, German Antimicrobial Resistance Strategy; DHMT, District Health Management Team; SOPs, standard operating procedures; KPIs, key performance indicators.

Sources: East African Community (34); Kenya Zoonotic Disease Unit (36); Federal Ministry of Health (16); Bayerlein and Villarreal (15); Government of Bangladesh (37).

Table 1 compares the institutional frameworks, thematic priorities, key achievements, implementation challenges, and governance lessons from the East African Community, Kenya, Germany, and Bangladesh. The comparison highlights differences in institutional maturity and identifies practical lessons that can inform Botswana’s implementation of an integrated One Health strategy for climate and health governance.

### 3.5 Limitations and critiques of the One Health approach

While the One Health approach is widely embraced as a transformative framework for tackling the complex connections between human, animal, and environmental health, it also faces significant limitations and criticism. As it becomes more prominent in policy discussions, global health strategies, and climate adaptation initiatives, scholars and practitioners have raised concerns about its conceptual clarity, implementation, and political implications. These critiques do not diminish the value of One Health; rather, they encourage its application in ways that are context-sensitive, inclusive, and responsive to issues of power, equity, and sustainability, particularly in low- and middle-income countries (LMICs) such as Botswana (31, 38, 39).

A key concern is the potential for technocratic dominance. As Craddock and Hinchliffe (38) observe, the increasing institutionalisation of One Health worldwide, often led by international agencies, funders, and scientific elites, can sideline local voices and grassroots perspectives. When initiatives are primarily designed and managed by technocrats or external consultants, there is a tendency to focus on biomedical or epidemiological approaches at the expense of social, cultural, and ecological considerations. This technocratic focus can lead to interventions that are scientifically valid but socially disconnected, failing to account for the realities, priorities, and knowledge systems of local communities. In rural Botswana, for example, where traditional ecological knowledge, pastoral practices, and local governance shape interactions between livestock and wildlife, overly top-down methods risk alienating key stakeholders and reducing programme success.

A related concern is the tendency to frame One Health primarily through the lens of health security and pandemic preparedness rather than broader objectives of health equity and environmental justice. This is especially clear in how some high-income countries and global health organizations frame One Health as a safeguard against zoonotic disease outbreaks “spilling over” from LMICs to the global North. Such narratives may unintentionally reinforce unequal global power relations by portraying LMICs primarily as sources of disease risk rather than as equal partners in prevention and innovation (25, 38). Securitizing One Health can also lead to narrow investments, such as bio-surveillance tools or quarantine facilities, at the expense of long-term health system development, ecological restoration, or community empowerment. For countries such as Botswana, where climate-related environmental change, endemic diseases, and health system capacity remain closely interconnected, a more balanced approach is needed that strengthens both health security and long-term system resilience.

Beyond these conceptual and political concerns, important implementation challenges continue to limit the routine operationalisation of One Health. While the idea of One Health is widely accepted, translating it into effective policies and sustained action remains inconsistent. Many national One Health strategies lack detailed implementation plans, realistic timelines, dedicated resource allocations, and clearly defined responsibilities across government sectors. For example, although Botswana recognises the importance of multisectoral collaboration in its national development and climate policy frameworks, there is limited evidence of sustained institutional coordination or dedicated budget allocations for One Health implementation. This implementation gap is further compounded by the absence of robust monitoring and evaluation frameworks capable of measuring effectiveness, efficiency, equity, and institutional performance over time. Without such mechanisms, tracking progress, holding institutions accountable, and supporting evidence-informed decision-making become considerably more difficult.

In low-resource settings, these challenges are worsened by systemic barriers such as limited funding, inadequate human resource capacity, and weak institutional infrastructure. Integrating One Health pillars such as joint surveillance, cross-sectoral governance, and community-based interventions requires not only conceptual agreement but also sustained logistical, technical, and political support. Many LMICs, including Botswana, face competing health priorities, dependence on external funding, and bureaucratic fragmentation, all of which can hinder the institutionalisation of One Health strategies (31, 40). For instance, fragmented budget lines across the health, agriculture, and environmental sectors can discourage collaboration. Similarly, heavy reliance on donor-funded projects, often operating on short funding cycles and externally determined priorities, can weaken national ownership and long-term planning.

Importantly, critiques of One Health also highlight its epistemological limitations. Although the approach is presented as transdisciplinary, it has been criticised for relying predominantly on Western biomedical paradigms while giving limited attention to Indigenous knowledge systems, critical social science perspectives, and decolonial approaches (38, 39). In many LMIC contexts, including Botswana, Indigenous communities possess rich ecological and ethno-veterinary knowledge that can strengthen surveillance, risk assessment, and health promotion. However, this knowledge is often overlooked within formal One Health programmes, reducing both the relevance and effectiveness of interventions. Recognising and integrating these knowledge systems requires more than methodological openness; it also demands institutional arrangements that enable meaningful community participation in planning, implementation, and evaluation while promoting epistemic justice and cultural humility.

Furthermore, the political economy of global health and environmental governance complicates the fair implementation of One Health. As several scholars have argued, the focus on specific diseases, regions, or interventions often reflects donor agendas, geopolitics, and market forces rather than local epidemiological or ecological realities (25, 31). This dynamic can bias One Health programmes toward highly visible or globally significant risks, such as emerging zoonoses, while neglecting endemic, climate-related, or non-communicable health issues that are more relevant to local communities. It also raises questions about who determines the priorities for One Health, whose interests benefit, and who is responsible for the outcomes. For Botswana, addressing these challenges will require adapting One Health implementation to national priorities, strengthening domestic ownership, and ensuring that governance arrangements respond to local institutional realities and community needs rather than external agendas alone. This reinforces the importance of nationally led governance systems that align international support with local priorities, strengthen institutional ownership, and promote long-term sustainability.

### 3.6 Relevance to Botswana’s health and climate governance

Botswana’s governance structures offer both opportunities and challenges for the institutionalisation of One Health strategies. The country enjoys political stability, relatively robust public institutions, and a track record of proactive public health interventions. However, the fragmentation of mandates across ministries such as Health, Agriculture, Environment, and Local Government creates siloed implementation environments that hinder integrated approaches. Bureaucratic inertia and sector-specific budget lines further limit coordinated action. The One Health approach offers a unifying paradigm that resonates with Botswana’s unique ecological and governance context, particularly its historical dependence on livestock, biodiversity-rich conservation areas, and rural public health infrastructure (5, 10). As climate variability increasingly affects water security, livestock production, biodiversity conservation, and public health, strengthening coordination across these sectors has become both a governance and a development priority (5, 10).

Integrating One Health into Botswana’s national development frameworks such as Vision 2036, National Development Plan 12 (NDP 12), and the National Climate Change Policy could foster transformative health outcomes. For instance, climate-related livestock diseases such as foot-and-mouth disease, vector-borne infections such as malaria, and antimicrobial resistance require institutional collaboration that bridges veterinary, environmental, and public health sectors (7, 10). Embedding One Health within these existing policy frameworks would also improve policy coherence by aligning climate adaptation, disaster risk reduction, biodiversity conservation, and health system resilience under a common governance framework (5, 8). The country’s relatively stable governance landscape, combined with growing research infrastructure through institutions such as the University of Botswana and the proposed National Public Health Institute, provides an opportunity to test multisectoral models with long-term sustainability (10).

Moreover, aligning One Health strategies with decentralised local governance could foster inclusive planning, particularly in Indigenous and remote communities vulnerable to climate impacts. District-level governance structures offer an important entry point for integrating surveillance, preparedness, and community engagement across sectors. Notably, the rural–urban divide in infrastructure and service delivery poses one of the greatest barriers to operationalising One Health in Botswana (9, 10). While urban centres benefit from better surveillance, laboratories, and human resources, rural areas are often left behind. To overcome these constraints, Botswana must strengthen interministerial coordination through mechanisms such as joint task forces, shared data platforms, and integrated budget lines. Capacity-building should be prioritised, especially at district and village levels, where One Health threats often emerge. Additionally, embedding One Health principles into national development frameworks can help mainstream these efforts across the policy landscape. Furthermore, this requires formalising the role of traditional leaders, community health workers, and local veterinarians in surveillance, preparedness, and response systems. Strengthening interoperability between veterinary surveillance, environmental monitoring, and public health information systems would further improve early warning and coordinated decision-making (6, 8).

Botswana’s participation in regional bodies such as SADC, the African Union, and Africa CDC also positions it well to champion harmonised regional strategies and pooled resource mobilisation. Rather than treating One Health as a donor-funded programme or a series of isolated pilot projects, Botswana should embed it within routine government planning, budgeting, monitoring, and performance management systems. Embedding One Health within core national planning and budgetary processes will strengthen institutional ownership, improve long-term sustainability, and build a more resilient health–environment interface capable of responding to present and future climate-related threats (5, 7, 10).

In rural Botswana, implementing One Health strategies is significantly constrained by systemic challenges. These include chronic shortages of qualified health professionals, particularly veterinarians and environmental health practitioners, limited infrastructure for diagnostics and surveillance, and weak internet or mobile coverage for digital monitoring. The vast geographic dispersion of communities and limited transportation networks further delay outbreak detection and coordinated responses. Addressing these constraints will require sustained investment in rural infrastructure, workforce development, digital surveillance systems, and community-based reporting mechanisms that strengthen the links between human, animal, and environmental health (5, 10). Such investments would not only improve preparedness for climate-sensitive diseases but also contribute to more equitable access to health services and greater resilience across Botswana’s health system.

## 4 Discussion and strategic implications for Botswana

The evidence gathered in this chapter shows that One Health advances from being just a concept to actual practice when it is legally mandated, funded through dedicated budgets, and embedded within routine governance through interoperable data systems and shared performance metrics (6, 7, 13, 42). Comparing different experiences reveals multiple pathways to institutionalisation: the East African Community’s regional platforms and national focal systems; Germany’s institutionalised antimicrobial resistance (AMR) governance under DART; and Bangladesh’s climate-responsive, community-based surveillance (16, 34, 37). Botswana’s case aligns with these lessons in principle because of its policy-driven governance tradition and strong veterinary and public health services, but implementation has been constrained by fragmented institutional mandates, sector-specific budgets, and inconsistent local surveillance capacity (9, 10). Collectively, these international experiences demonstrate that institutionalising One Health is fundamentally a governance reform requiring sustained political commitment, organisational coordination, and long-term investment rather than a series of isolated technical interventions (5, 8).

Three broad lessons emerge from this analysis, each of which has important implications for strengthening One Health implementation in Botswana.

First, the governance lesson is that clarity of mandate matters more than the breadth of aspiration. The EAC model shows that multisectoral collaboration is sustained when a platform has clear terms of reference, escalation procedures, and a standing secretariat that brings together health, livestock, environment, and disaster management on a regular basis (34). Botswana’s existing coordination mechanisms could be strengthened through a National One Health Platform established under an appropriate legal or policy instrument, with the secretariat located within the Department of Public Health to coordinate surveillance and emergency preparedness. This would also establish an interface with SADC mechanisms for cross-border threats, aligned with the regional cooperation framework that underpins One Health (7, 13). Embedding clear accountability arrangements and routine reporting requirements within this platform would further strengthen institutional continuity and reduce dependence on informal collaboration (6).

Second, the institutional lesson is that financing and metrics must be integrated, not added on. Germany’s DART shows how inter-agency collaboration becomes standard when linked to legal reporting, measurable indicators, and stable budget lines that span ministries, including environmental sectors such as wastewater and agriculture (16). In Botswana, incorporating One Health indicators into the Health Sector Performance Matrix with traffic-light (green/amber/red) signals for AMR, zoonotic preparedness, and climate-sensitive risks would support Vision 2036’s governance and service excellence pillar while allowing NDP 12 to treat One Health as a cross-cutting strategy rather than a standalone project (5, 10). A modest, dedicated budget for multisectoral One Health activities would further strengthen implementation by supporting joint planning, surveillance, workforce development, and routine preparedness across sectors (7). Routine monitoring of these indicators would also provide an evidence base for policy learning, adaptive management, and future resource allocation while strengthening transparency and institutional accountability (6).

Third, the operational lesson is to create systems that reduce risk before crises escalate. Bangladesh’s experience shows that community event-based detection, validated at the district level and combined with environmental and veterinary signals, shortens the time between detecting a signal and responding. For Botswana, strengthening coordination among District Health Management Teams, Livestock Advisory Centres, and Village Development Committees through a shared reporting network supported by simple digital tools would help close the rural–urban surveillance gap and improve the early detection of vector shifts, animal die-offs, and water quality threats (6, 37). This approach also addresses critiques that One Health can become too technocratic or securitised by refocusing on community knowledge and participation as central elements of risk management (8, 38). Strengthening interoperability between environmental monitoring, veterinary surveillance, and public health information systems would further improve the country’s capacity to anticipate and respond to climate-sensitive health threats while supporting integrated decision-making across sectors (6, 8).

Building on these governance, institutional, and operational lessons, four strategic policy directions emerge for Botswana’s current planning cycle.

The first strategic direction is to consolidate the legal and institutional framework by establishing a National One Health Platform through an appropriate legal or policy instrument, which would mandate data sharing, joint investigation protocols, and clear escalation pathways. This platform should be connected to SADC coordination for cross-border events and aligned with broader regional One Health governance arrangements (7, 13, 34).

The second strategic direction is to strengthen financing and accountability by allocating a modest but recurring One Health budget to support joint simulations, integrated training, laboratory–environment interfaces, and community surveillance. A concise set of One Health indicators should also be incorporated into annual performance reviews using a traffic-light reporting model to strengthen transparency, accountability, and adaptive management (6, 7, 16).

The third strategic direction is to strengthen local surveillance and stewardship by investing in district-level capacity, training community animal health workers and environmental officers to report unusual events and integrating these data into national surveillance and early warning systems through the National Public Health Institute (6, 9). Community participation should be recognised as a strategic governance asset that strengthens trust, improves surveillance quality, and enhances the legitimacy of public health responses.

The fourth strategic direction is to align One Health with national development and climate strategies by explicitly embedding it within NDP 12, Vision 2036, universal health coverage reforms, and national climate policy. This would help ensure that interventions addressing antimicrobial resistance, food safety, vector control, ecosystem restoration, and climate adaptation reinforce one another rather than compete for fragmented resources (1, 5, 7, 10).

Positioning Botswana within regional and global governance completes the picture. Regionally, a legally anchored platform and transparent indicator set would enable Botswana to contribute to SADC-wide harmonisation of AMR surveillance, pooled veterinary and public health laboratory standards, and cross-border event-based reporting with neighbouring states (5, 7). Continent-wide, alignment with the Africa CDC One Health Strategy would position Botswana among countries helping to shape operational guidance rather than simply adopting it, creating opportunities for technical exchange, regional learning, and resource mobilisation (41). At the global level, Botswana is well positioned to contribute practical evidence on implementing One Health in middle-income African settings, particularly in relation to climate resilience, integrated governance, and community-based surveillance. Such engagement would strengthen knowledge exchange while ensuring that Botswana both benefits from and contributes to the continued evolution of the global One Health agenda (5, 7).

The strategic goal is not to introduce another vertical initiative but to embed One Health as a governance principle integrated into law, budgets, performance systems, and routine institutional practice. Achieving this within NDP 12 and Vision 2036 would simultaneously strengthen universal health coverage, climate resilience, and public trust while positioning Botswana as a regional example of context sensitive One Health implementation (1, 5, 10). Ultimately, the long-term success of One Health in Botswana will depend not only on technical capacity but also on sustained political leadership, institutional commitment, predictable domestic financing, and meaningful collaboration across sectors and communities (6, 7).

In conclusion, the One Health approach should be viewed not only as an ethical imperative but also as a strategic investment in Botswana’s future resilience. As climate change, biodiversity loss, and emerging health threats become increasingly interconnected, governance systems must evolve accordingly. The central message of this chapter is that the One Health approach should not be treated as another stand-alone programme but as a core principle of public sector governance embedded within legislation, financing, surveillance, and multisectoral coordination. By institutionalising this approach within routine government systems, Botswana has an opportunity to strengthen climate resilience, protect population health, and provide a practical model for integrated One Health implementation across Africa.
